# Correction to “Italian Delphi Consensus on Maternity Care in Multiple Sclerosis: Toward an Interdisciplinary Model of Care”

**DOI:** 10.1111/ene.70801

**Published:** 2026-10-28

Portaccio E , Cavalla P , Moiola L , Patti F , Marozio L , Palma P , Campobasso M , Balzani F , Villani A , Barbiero VS , Facchinello D , Robello R , Marfia GA ; Maternity Care Delphi Expert Panel . Italian Delphi Consensus on Maternity Care in Multiple Sclerosis: Toward an Interdisciplinary Model of Care. European Journal of Neurology 33, no. 8 (2026): e70700, 10.1111/ene.70700.PMC1344556542559795

In Appendix A to the article, the name “Francesco Perini, Neurology Clinic—University Hospital of Padua, Padua, Italy” was incorrect. This should have read “Paola Perini, Neurology Clinic—University Hospital of Padua, Padua, Italy.”

We apologize for this error.

